# Supplementary material for: Salt Priming as a Smart Approach to Mitigate Salt Stress in Faba Bean (Vicia faba L.)
Source: Plants (Basel). 2022 Jun 20;11(12):1610. doi: 10.3390/plants11121610 (PMC9228577; doi:10.3390/plants11121610)
Supplement: Supplementary file 1 [file plants-11-01610-s001.zip › plants-1763446-supplementary.pdf]

Supplementary data

Table S1: Effect of different salt treatments (NC: negative control, PC: positive control, P: salt priming, Shk: salt shock, and P/Shk: salt priming then salt shock) on shoot fresh weight, shoot dry weight, root fresh weight, root dry weight, leaf area, plant height, pod length, pods weight/plant, the weight of 1000 seeds, seed yield/plant of faba bean plants and membrane stability index. Growth traits were recorded at 58 DAS while yield was recorded at 100 DAS (days after sowing). The values represented in the figure indicate the mean of 3 replicates (±SE). Different letters on the error bars indicate significant differences among salt treatments ($p < 0.05$).

**Parameter**	**At the end of salt shock period (100 DAS)**				
	NC	PC	P	Shk	P/Shk
**Shoot FW**	22.2^{a}±0.88	16.8^{c}±0.03	21.7^{a}±0.9	17.9^{bc}±0.1	19.3^{b}±0.29
**Shoot DW**	2.64^{a}±0.12	11.4^{d}±0.05	2.16^{b}±0.07	1.8^{c}±0.02	2.1^{b}±0.02
**Root FW**	23.69^{b}±0.48	16.09^{c}±0.76	27.23^{a}±0.64	20.3^{c}±0.45	25.4^{b}±0.55
**Root DW**	1.76^{c}±0.09	1.09^{d}±0.07	2.33^{a}±0.3	1.8^{c}±0.04	2.0^{b}±0.02
**Leaf area**	46.15^{a}±2.26	34.43^{c}±1.73	42.13^{ab}±1.21	32.2^{c}±0.56	39.7^{b}±0.47
**Plant height**	32.67^{a}±0.6	26.33^{c}±0.44	30.17^{b}±0.44	2.3.8^{d}±0.6	27.2^{c}±0.6
**Pod length**	10.5^{a}±0.29	6.33^{d}±0.17	9.67^{b}±0.17	8.5^{c}±0.26	9.5^{b}±0.09
**Pod wt/plant**	18.01^{a}±0.16	9.36^{c}±0.68	17.71^{a}±0.03	15.1^{b}±0.54	16.8^{a}±0.35
**Weight of 1000 seeds**	1.46^{a}±0.02	0.89^{d}±0.03	1.37^{b}±0.03	1.1^{c}±0.03	1.33^{b}±0.02
**Seed wt/plant**	9.62^{a}±0.36	4.71^{d}±0.57	8.56^{ab}±0.21	7.3^{c}±0.07	8.5^{b}±0.26
**MSI**	80^{b}±0.3	74.85^{c}±0.5	81.89^{a}±0.9	72.24^{d}±0.2	79.67^{b}±0.3

Table S2: Effect of different salt treatments (NC: negative control, PC: positive control, P: salt priming, Shk: salt shock, and P/Shk: salt priming then salt shock) on total chlorophyll, carotenoids, and carotenoids/total chlorophyll ratio, proline content, total soluble sugars content, MDA level, H_2O_2 content, free and total phenolics. The values represented in the figure indicate the mean of 3 replicates (±SE). Different letters on the error bars indicate significant differences among various salt treatments ($p < 0.05$).

**Parameter**	**At the end of priming period (46 DAS)**			**At the end of salt shock period (58 DAS)**				
	NC	PC	P	NC	PC	P	Shk	P/Shk
**Total chl**	1.11^{a}±0.06	0.89^{b}±0.04	1.11^{a}±0.05	0.85^{a}±0.03	0.73^{b}±0.02	0.79^{ab}±0.01	0.61^{c}±0.01	0.8ab±0.02
**Carotenoids**	0.61^{a}±0.01	0.45^{b}±0.02	0.51^{b}±0.02	0.40^{a}±0.02	0.38^{a}±0.01	0.41^{a}±0.01	0.29^{b}±0.02	0.4^{a}±0.01
**Total chl/Caro**	0.55^{a}±0.01	0.51^{b}±0.01	0.46^{c}±0.01	0.47^{a}±0.01	0.52^{a}±0.02	0.52^{a}±0.01	0.48^{c}±0.01	0.49^{b}±0.01
**Proline**	1.31^{c}±0.01	2.28^{b}±0.03	3.00^{a}±0.01	2.22^{e}±0.2	3.67^{d}±0.2	4.9^{c}±0.12	5.73^{b}±0.34	6.37^{a}±0.12
**T.S.S.**	4.87^{b}±0.26	6.9^{a}±0.39	7.77^{a}±0.49	6.79^{b}±0.04	2.61^{c}±0.04	8.54^{a}±0.49	5.75^{b}±0.37	5.01^{b}±0.37
**MDA**	16.9^{c}±0.14	25.13^{a}±0.47	20.03^{b}±0.59	17.7^{d}±0.47	26.9^{a}±0.48	20.23^{c}±0.69	26.07^{a}±1.72	26.64^{b}±0.55
**H_2O_2**	0.17^{c}±0.02	0.26^{a}±0.01	0.23^{b}±0.01	0.17^{c}±0.02	0.30^{a}±0.01	0.32^{b}±0.01	0.29^{a}±0.01	0.23^{b}±0.01
**Free phenolics**	9.82^{c}±0.28	10.47^{b}±0.05	13.75^{a}±0.05	9.8^{c}±0.15	10.39^{c}±0.15	16.02^{b}±0.22	16.85^{b}±0.2	19.93^{a}±0.57
**Total phenolics**	16.55^{c}±0.33	21.72^{b}±0.17	425.39^{a}±0.72	18.48^{d}±0.35	21.96^{c}±0.35	24.63^{b}±0.27	26.16^{a}±0.34	21.96^{c}±0.35

Table S3: Effect of different salt treatments (NC: negative control, PC: positive control, P: salt priming, Shk: salt shock, and P/Shk: salt priming then salt shock) on POX, CAT, GR, APX, SOD activities (U min^{-1} mg^{-1} protein) and on qPCR gene expression of glutathione and ascorbate metabolism genes: *L-APX*; L-ascorbate peroxidase, *LAP*; leucyl aminopeptidase, *SPS*; spermidine synthase, *AP-N*; aminopeptidase N, and *RDR-M*; ribonucleoside-diphosphate reductase subunit M1 gene. The values represented in the figure indicate the mean of 3 replicates (±SE). Different letters on the error bars indicate significant differences among various salt treatments ($p < 0.05$).

**Parameter**	**At the end of priming period (46 DAS)**			**After first seven hrs of salt shock period (51 DAS)**					**At the end of salt shock period (58 DAS)**				
	NC	PC	P	NC	PC	P	Shk	P/Shk	NC	PC	P	Shk	P/Shk
**APX activity**	0.76^{c}±0.09	1.06^{b}±0.04	1.68^{a}±0.06	1.36^{d}±0.03	3.02^{b}±0.13	2.53^{c}±0.12	2.84^{b}±0.11	3.34^{a}±0.12	0.86^{d}±0.13	1.30^{c}±0.02	1.95^{b}±0.13	2.15^{b}±0.14	2.81^{a}±0.18
**GR activity**	0.58^{c}±0.04	0.85^{b}±0.03	1.05^{a}±0.02	0.52^{c}±0.03	0.73^{b}±0.03	0.86^{a}±0.04	0.80^{ab}±0.05	0.95^{a}±0.05	0.57^{c}±0.07	1.05^{b}±0.03	1.08^{b}±0.06	1.10^{b}±0.03	1.19^{a}±0.03
**SOD activity**	1.73^{b}±0.11	2.55^{a}±0.09	2.62^{a}±0.04	1.57^{c}±0.05	2.18^{b}±0.05	2.59^{a}±0.06	2.77^{a}±0.09	2.70^{a}±0.09	1.70^{c}±0.12	3.57^{a}±0.05	3.25^{b}±0.1	3.31^{b}±0.05	3.16^{b}±0.05
**POX activity**	1.40^{b}±0.08	2.53^{a}±0.05	2.62^{a}±0.02	1.18^{d}±0.1	2.25^{c}±0.02	2.75^{b}±0.08	3.30^{a}±0.03	3.06^{a}±0.14	1.05^{c}±0.01	2.16^{b}±0.01	2.40^{b}±0.2	3.47^{a}±0.07	2.67^{b}±0.04
**CAT activity**	0.19^{c}±0.02	0.27^{b}±0.01	0.42^{a}±0.06	0.21±^{e}0.01	0.33^{d}±0.02	0.49^{c}±0.04	0.54^{b}±0.07	0.70^{a}±0.02	0.34^{e}±0.04	0.76^{b}±0.01	0.63^{d}±0.04	0.84^{a}±0.02	0.71^{c}±0.03
***L-APX* gene**	1^{c}±0.03	1.45^{b}±0.02	1.7^{a}±0.09	1^{d}±0.05	2.31^{b}±0.06	1.59^{c}±0.05	2.85^{a}±0.07	2.15^{b}±0.03	1^{d}±0.06	1.57^{c}±0.06	2.25^{b}±0.05	2.58^{a}±0.04	1.46^{c}±0.04
***SpS* gene**	1^{c}±0.02	1.45^{b}±0.04	3.07^{a}±0.02	1^{c}±0.04	1.69^{b}±0.02	1.53^{b}±0.05	1.94^{a}±0.05	1.2^{c}±0.04	1^{d}±0.05	1.47^{c}±0.02	4.26^{a}±0.04	2.76^{b}±0.02	1.37^{c}±0.06
***LAP* gene**	1^{c}±0.02	1.59^{b}±0.05	4.32^{a}±0.03	1^{c}±0.03	1.34^{a}±0.03	1.42^{a}±0.03	1.09^{c}±0.06	1.18^{b}±0.05	1^{d}±0.04	1.2^{c}±0.04	1.29^{b}±0.04	1.77^{a}±0.07	1.13^{c}±0.05
***AP-N* gene**	1^{c}±0.02	1.8^{b}±0.06	4.66^{a}±0.05	1^{d}±0.03	1.98^{b}±0.02	2.28^{a}±0.02	2.12^{a}±0.07	1.5^{c}±0.04	1^{d}±0.03	4.05^{c}±0.05	5.99^{b}±0.04	7.16^{a}±0.05	3.87^{c}±0.05
***RDS-M* gene**	1^{c}±0.02	1.3^{b}±0.03	2.48^{a}±0.06	1^{c}±0.02	1.73^{b}±0.05	1.87^{b}±0.06	2.22^{a}±0.02	1.75^{b}±0.08	1^{e}±0.04	2.19^{c}±0.06	3.91^{a}±0.02	1.4^{d}±0.06	3.42^{b}±0.04

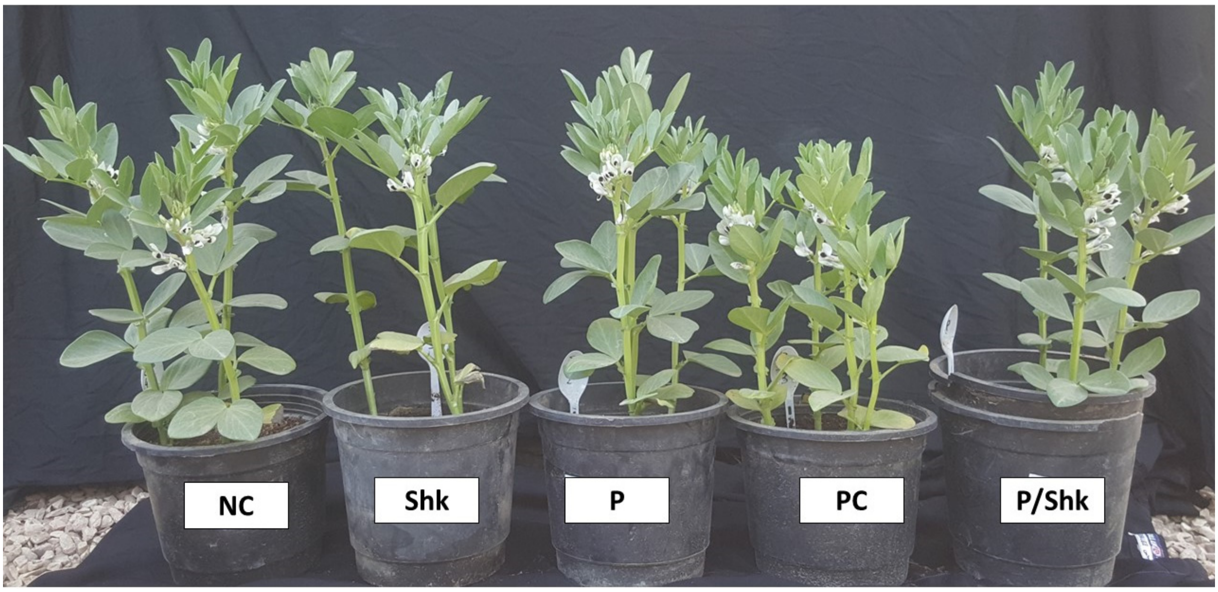

Figure S1: Effect of salt treatments on Sakha1 plant growth after 58 DAS.
